# Supplementary material for: A protocol for the co-creation and usability/acceptability testing of an evidence-based, patient-centred intervention for self-management of urinary incontinence in older men
Source: PLoS One. 2024 Aug 12;19(8):e0306080. doi: 10.1371/journal.pone.0306080 (PMC11318901; doi:10.1371/journal.pone.0306080)
Supplement: S1 Appendix — (PDF) [file pone.0306080.s001.pdf]

Appendix

Appendix 1:

Self-management intervention/tool usability and acceptability survey

Product usability scale

Please check the box that reflects your immediate response to each statement. Don't think too long about each statement. Please make sure you respond to every statement. If you don't know how to respond, simply check box "3."

	Strongly Disagree			Strongly Agree	
1. I think that I would like to use this tool/app frequently	1	2	3	4	5
2. I found the tool/app unnecessarily complex	1	2	3	4	5
3. I thought the tool/app was easy to use	1	2	3	4	5
4. I think that I would need the support of a technical person to be able to use this tool/app	1	2	3	4	5
5. I found the various functions in the tool/app were well integrated	1	2	3	4	5
6. I thought there was too much inconsistency in this tool/app	1	2	3	4	5
7. I imagine that most people would learn to use this tool/app very quickly	1	2	3	4	5
8. I found the tool/app very awkward to use	1	2	3	4	5
9. I felt very confident using the tool/app	1	2	3	4	5
10. I needed to learn a lot of things before I could get going with this tool/app	1	2	3	4	5

11. Overall, I would rate the user-friendliness of this product as:

□□<br><br>Worst imaginable	□□<br><br>Awful	□<br><br>Poor	□□<br><br>OK	□□<br><br>Good	□□<br><br>Excellent	□□<br><br>Best imaginable

Acceptability survey (satisfaction with the intervention)

1. How satisfied were you with the intervention?
    a. Very satisfied
    b. Somewhat satisfied
    c. Neither satisfied nor dissatisfied
    d. Somewhat dissatisfied
    e. Very dissatisfied

2. What did you like best about the intervention?

----

3. Can you identify any disadvantages or limitations of the intervention?
