# Supplementary material for: A protocol for the co-creation and usability/acceptability testing of an evidence-based, patient-centred intervention for self-management of urinary incontinence in older men
Source: PLoS One. 2024 Aug 12;19(8):e0306080. doi: 10.1371/journal.pone.0306080 (PMC11318901; doi:10.1371/journal.pone.0306080)
Supplement: Attachment — Submitted filename: Response to reviewers.docx [file pone.0306080.s002.docx]

RESPONSE LETTER

Dear Reviewers,

We appreciate your comments and the opportunity to improve our manuscript. This table shows our response and the pages where changes have been made to the manuscript.

Sincerely,

Olawunmi Olagundoye (On behalf of the authors)

| Reviewer | Original comments of the reviewer | Reply | Changes done on page number, paragraph or line number |
| --- | --- | --- | --- |
| **Reviewer 1** | Reviewer #1: The protocol provides the grounding for what looks to be a robust and interesting study, with a clear methodology. I just have a couple of minor suggestions which may strengthen it further  The introduction refers to only 22% of men seeking assistance- is this just in high income countries or do we know if this is a global figure that also takes into account low- and middle income countries? It would be good to clarify this.  Similarly in the same paragraph, it may be useful to name some examples of contexts in which men hold significant societal (even traditional) roles. | Your suggestions are greatly appreciated. Thank you.    A clarification specifying the study population and study location responsible for the quoted figure (22% of men) has been added to the introduction.    Some examples of men's significant societal roles have been added. | Page 3, second paragraph  Page 3, last paragraph |
|  | In the section on urinary incontinence interventions on page 11, there is a line stating how older people perceived UI as a part of aging. Is this from a particular research study? Better context for this study needs to be added- which older people? Where? | The context has been added. | Page 4, paragraph 3 |
|  | On page 13 I had a couple of minor comments. The authors could add 1-2 examples of the stakeholders they plan to engage in the intervention mapping etc.  The paper rightly highlights the lack of focus on older men. A couple of references and examples could be added to highlight the existing significant focus on older women, just for context and to further emphasise this significant gap. In the same paragraph, should the paper be referring to 'health seeking behaviours' rather than just health seeking? | The proposed study will involve continence experts and older men with UI, as described in the section on the formation of the participatory group.  This has been done.  It has been changed to “health seeking”. | Page 10, from the second to the last paragraph to page 11, first paragraph.  Page 4, first paragraph  Abstract, first paragraph |
|  | Finally on page 17. How will recruitment take into account diversity, are there any specific efforts being made to include men from ethnic minorities/First Nations or indigenous groups?  This is a clear protocol for a promising study. | We will recruit ethnically diverse older men into the co-creation group through existing ethnically diverse patient advisory boards, seniors' clubs, and ethnic associations.  Thank you very much for your feedback. | Page 11, first paragraph |
| **Reviewer 2** | This is a well-written manuscript focusing on a sadly under-addressed area of key importance in public health, incontinence in older males. The manuscript describes a study that will be undertaken to gain understanding in how self-management for UI in elderly males can be developed. The literature review demonstrates a deep understanding of the subject area and relates to well posed aim and objective. The methods are appropriate and show a keen appreciation of how clinical research, particularly in stigmatised subjects such as incontinence, should be conducted.  Very minor ammendments are recommended, as outlined below - which do not warrant re-review. Hence a recommendation of accept. Excellent and commendable research. | Your feedback is greatly appreciated. Thank you very much. |  |
|  | Abstract: Usability/acceptability testing - please rephrase to avoid use of / in the text. Can you briefly define 'logic model' as part of the intervention mapping (IM) framework - to make the abstract more accessible to a wider audience? E.g. the logic-model (which defines the ...) | We have removed the slash and replaced it with and/or, so as to reflect what may happen in the study based on participants' preferred methods of intervention delivery.  The logic model has been defined in simple terms for the benefit of a wider audience. Thank you. | Page 2, second and third paragraph, page 10, first paragraph, page 13, first and last paragraphs  Page 10, last paragraph, page 11, last paragraph |
|  | In the main body - please re(define) IM, (not just include in the abstract) and provide a short defintion to make the paper self-contained. | This has been done.  Thank you. | Page 10, second paragraph |
